# Purification of retinal ganglion cells using low-pressure flow cytometry

**DOI:** 10.3389/fnmol.2023.1149024

**Published:** 2023-07-20

**Authors:** Kiran J. Mcloughlin, Afnan M. Aladdad, Andrew J. Payne, Anna I. Boda, Sayra Nieto-Gomez, Karl E. Kador

**Affiliations:** ^1^Department of Ophthalmology, School of Medicine, University of Missouri-Kansas City, Kansas City, MO, United States; ^2^Department of Biomedical Sciences, School of Medicine, University of Missouri-Kansas City, Kansas City, MO, United States

**Keywords:** retinal ganglion cell (RGC), FACS (fluorescence-activated cell sorting), immunopanning, cell isolation, neurite outgowth

## Abstract

Purified Retinal Ganglion Cells (RGCs) for *in vitro* study have been a valuable tool in the study of neural regeneration and in the development of therapies to treat glaucoma. Traditionally, RGCs have been isolated from early postnatal rats and mice, and more recently from human *in vitro* derived retinal organoids using a two-step immunopanning technique based upon the expression of Thy-1. This technique, however, limits the time periods from which RGCs can be isolated, missing the earliest born RGCs at which time the greatest stage of axon growth occurs, as well as being limited in its use with models of retinal degeneration as Thy-1 is downregulated following injury. While fluorescence associated cell sorting (FACS) in combination with new optogenetically labeled RGCs would be able to overcome this limitation, the use of traditional FACS sorters has been limited to genomic and proteomic studies, as RGCs have little to no survival post-sorting. Here we describe a new method for RGC isolation utilizing a combined immunopanning-fluorescence associated cell sorting (IP-FACS) protocol that initially depletes macrophages and photoreceptors, using immunopanning to enrich for RGCs before using low-pressure FACS to isolate these cells. We demonstrate that RGCs isolated via IP-FACS when compared to RGCs isolated via immunopanning at the same age have similar purity as measured by antibody staining and qRT-PCR; survival as measured by live dead staining; neurite outgrowth; and electrophysiological properties as measured by calcium release response to glutamate. Finally, we demonstrate the ability to isolate RGCs from early embryonic mice prior to the expression of Thy-1 using Brn3b-eGFP optogenetically labeled cells. This method provides a new approach for the isolation of RGCs for the study of early developed RGCs, the study of RGC subtypes and the isolation of RGCs for cell transplantation studies.

## Introduction

Injury of the retinal ganglion cells (RGCs) which ultimately leads to cell death is the primary cause of blindness in the disease of glaucoma. As a result, these cells have been widely used in the study of the disease mechanism as well as in the development of neuroprotective and neuroregeneration treatments (Wang et al., 2007). In addition to their use in the study of glaucoma, RGCs have been widely used for several decades in the study of the central nervous system (CNS) as a whole, both *in vitro* in purified cell cultures and *in vivo* using optic nerve crush studies. This role in CNS injury and regeneration research is due to the RGCs being the most accessible CNS projection neuron, their ability to extend neurites *in vitro* following isolation through an early postnatal age, and the ability of the cells to be isolated and purified to an extremely high purity (>99%) (Barres et al., 1988).

Isolation of RGCs from rodents has been performed by immunopanning (Barres et al., 1988), antibody conjugated magnetic beads (Gill et al., 2016) and fluorescence activated cell sorting (FACS) (Chintalapudi et al., 2017), with each of these methods using a two-step purification based upon the immunoreactivity of Thy-1 (CD90). While each of these methods is based on the same immunoreactivity, it has previously been shown that the purity and survival of the isolated RGCs is variable between the three procedures (Gao et al., 2016). Immunopanning has been reported to purify RGCs to 99.5% purity, based upon retrograde labeling placed in the brain and with a survival of up to 95%; however, this purity appears to be dependent on the Thy-1 antibody used and the proficiency of the laboratory doing the isolation with other labs reporting a purity closer to 80%. Isolation of RGCs using the magnetic bead separation has reported at 95% purity, with RGCs having a similar short-term survival to immunopanned RGCs; however, magnetically separated cells died in experiments after 9 days whereas immunopanned RGCs were viable for over 3 weeks. FACS separation of RGCs was able to purify RGCs to nearly 100%; however, these cells were not viable in short term experiments following separation and as a result this method has primarily been used in genomic and proteomic analysis experiments where further culture of the RGCs does not occur. This lack of viability is thought to be related to the high pressures required during the separation, which has been shown to alter gene expression and cause oxidative stress in other cell types (Llufrio et al., 2018).

While utilized in each of these methods, basing RGC isolation upon Thy-1 reactivity limits the developmental stage at which the cells can be purified, missing early development and axon extension which occurs prior to Thy-1 expression at rat embryonic day 17, and limiting the effectiveness of the purification of adult RGCs as Thy-1 expression decreases throughout adulthood (Schmidt et al., 1995; Liu et al., 1996; Wang et al., 2007). Most importantly, these protocols are also limited in their usefulness of isolating RGCs from injury models, as Thy-1 is down regulated after injury (Huang et al., 2006). Other markers, both for all types of RGCs and for specific subtypes of these cells do exist, but these are primarily intracellular markers or are shared with a subset of other retinal neurons (Badea and Nathans, 2011; Rodriguez et al., 2014; Groman-Lupa et al., 2017; Langer et al., 2018; Miltner et al., 2019), and as a result will result in a mixed cell population or would not be compatible with immunopanning or magnetic bead separation methods.

Recently, new technologies which utilize microfluidics have been developed for cell sorting which utilize lower pressures for their separation, less than 2 psi, compared to standard cell sorters, which require 20 psi for their separation (Chen et al., 2009; Jagnandan and Morachis, 2022). In these systems, cells can be separated using label free properties such as size or shape or through the use of fluorescent labeling similar to more traditional FACS purification (Cho et al., 2010; Munoz and Morachis, 2022). These systems have been shown to be compatible with cells that are more fragile, such as stem cells and neurons.

This study aims to utilize a low-pressure cell sorting platform to isolate RGCs from a dissociated retinal cell suspension. In this study we will isolate RGCs from early postnatal rats using fluorescently labeled antibodies against Thy-1 and then compare their purity, survival, outgrowth potential and their electrophysiological ability to RGCs isolated via the immunopanning method. We will also evaluate whether RGC isolation via cell sorting can be enhanced by using a separate immunopanning step to remove CD73 positive immature photoreceptors (Quintero et al., 2016). Finally, we will evaluate the ability of low-pressure FACS to isolate optogenetically labeled RGCs from the Brn3b-GFP mouse at embryonic day 14–15, an age that is prior to the expression of Thy-1. We hypothesized that there will be no discernible differences between RGCs isolated via immunopanning and those isolated via the low-pressure FACS method, providing a new tool for RGC isolation not possible using previously developed methods.

## Materials and methods

All procedures conformed to institutional animal care and use authorizations and were in accordance with regulations established by the National Institutes of Health and the ARVO Statement for the Use of Animals in Ophthalmic and Vision Research.

### Postnatal retinal ganglion cell isolation and culture

RGCs were purified by two methods, immunopanning and a combined immunopanning low-pressure FACS sorting method. In both methods, retinas were dissected from postnatal day 2 Sprague Dawley rat litters (10–16 pups per experiment), digested using papain (Sigma Aldrich), and dissociated into single cells using mechanical trituration. Following negative selection to remove macrophages and endothelial cells via immunopanning (anti-rat macrophage, Accurate Chemical and Scientific, AIA51240), the dissociated retinal suspension was divided into two parts: one to proceed with the standard immunopanning method and the second to follow the low-pressure cell sorting method.

For the immunopanning method, the retinal cell suspension was incubated with a Thy-1 adsorbed petri dish for 50 min for RGC selection. Following selection, the petri dish was washed and the RGCs isolated via trypsin release. RGCs were resuspended in RGC growth media composed of neurobasal media (Gibco, Life Technologies) supplemented with insulin, N-acetyl cysteine, triiodo-thyronine, forskolin (Sigma Aldrich), penicillin/streptomycin, GlutaMax (Gibco, Life Technologies), sodium pyruvate, B27 (Thermo Fisher Scientific), and BDNF and CNTF growth factors (PEPROTECH), at previously published concentrations and plated as described below (Kador et al., 2013). For cultures lasting longer than 3 days, half media changes were conducted every second day.

The second half of the retinal suspension was centrifuged at 80 x G for 15 min before resuspending in PBS supplemented with 0.02% insulin +0.04% BSA (herein called panning buffer) at a density of 1 × 10^7^ cells per mL. The cell suspension was then incubated with 2.5 μg mouse anti-rat anti-CD90.1-FITC (BD Biosciences, Franklin Lakes, NJ, United States) per 1 × 10^7^ cells for 20 min at room temperature and protected from the light. Cells were then washed in panning buffer and again centrifuged at 80 x G for 10 min before resuspension at a final density of 1 × 10^6^ cells per mL in the panning buffer. CD90.1-positive sorting was performed on WOLF Benchtop Microfluidic Cell Sorter (Nanocellect, San Diego, CA, United States) at a rate of 300 cells/s with panning buffer being used as both the running buffer and the sheath fluid. An unstained control was prepared in tandem to calibrate the instrument before sorting. The instrument was calibrated before use using manufacturer-provided calibration beads, and unstained control cells were used to determine gating controls. Isolated cells were finally centrifuged and resuspended in the RGC growth media described above.

### Embryonic retinal ganglion isolation

Embryonic day 14–15 RGCs were isolated from homozygous Brn3b-P2A-eGFP transgenic mice created by the Animal Modeling Core Facility at the University of Missouri- Columbia crossed with CD-1 female mice. Mating pairs were maintained for 48 h and females sacrificed 15 days following pairing. Embryonic retinas were isolated and the retinas digested using papain and broken down to single cells using mechanical trituration, following the same procedure as postnatal RGC isolation. The retinal cell suspension was then incubated with anti-macrophage antibody for the removal of macrophage and endothelial cells, and anti-CD73 coated petri dishes for the isolation and removal of embryonic photoreceptors, respectively. The cell concentration of the unbound retinal digestion was adjusted to 1 × 10^6^ cells per mL in panning buffer and separated based upon endogenous eGFP fluorescence using the Wolf Cell Sorter. RGCs were plated and cultured as above.

### Substrates coating and seeding

24-glass-bottom well plates were used for viability and immunocytochemistry, 35 mm MatTek glass-bottom dishes were used for Ca^2+^ imaging, and 6-well plates were used for RNA extraction experiments. All samples were coated with poly-D-lysine (PDL, 1 mM, Sigma) for 30 min, washed with 1x PBS, and coated with Neurobasal media containing laminin (2 μg/mL, Thermo Fisher Scientific) for 24 h. Substrate surfaces were then washed and pre-incubated in culture media before seeding. Cells were seeded on substrates at a density of 6,500 cells/cm^2^ for calcium imaging experiments, 1,000 cells/cm^2^ for viability experiments, and 1,000 cells/cm^2^ for tracing experiments. All samples were cultured at 37°C in 10% CO_2_ in air and 100% humidity.

### Determination of RGCs viability using calcein-AM and propidium iodide staining

Calcein-AM and propidium iodide staining was utilized for simultaneous fluorescence staining of viable (calcein-AM) and dead (propidium iodide) cells and was used as an endpoint assay. Ten microliter of calcein-AM and 5 μL of propidium iodide (both from Thermo Fisher Scientific) were added to 5 mL of RGC growth media. Following 3, 7, or 14 days in culture, seeded cells were incubated with 500 μL of the assay solution for 15 min at 37°C before being imaged using a Leica DMI 6000 inverted fluorescent microscope. As a positive control for propidium iodide, RGCs which had been fixed with 4% (w/v) paraformaldehyde (Electron Microscopy Systems) were incubated using the same solution and were used to set the red propidium iodide exposure setting during imaging.

### Immunocytochemical staining

Following 3 days in culture, cells were fixed using 4% (w/v) paraformaldehyde for 30 min. Blocking was performed for 30 min at RT in PBS with 10% (v/v) goat serum and 0.2% Triton-X100. Samples were incubated with primary antibodies of mouse anti-beta-III Tubulin (βIII-tubulin/RGCs marker: 1:500, Abcam) and rabbit anti-RNA-binding protein (Rodriguez et al., 2014) with multiple splicing (anti-RPBMS/RGCs marker: 1:500, Abcam) at 4°C overnight. After washing the samples with PBS, they were incubated with secondary antibodies of goat anti-mouse IgG Alexa Fluor 488 (1:500) and goat anti-rabbit IgG Alexa Fluor 647 (1:500) (Abcam) at 4°C overnight. Samples were washed once with PBS for 5 min and then incubated for 5 min with DAPI (1:2500 in PBS), followed by two 5 min washes with PBS. Images were obtained using an EVOS™ Fluorescence Auto 2 microscope by imaging the central 60% of each well. Using ImageJ, cell neurites were traced to quantify average neurite length, total neurite length, longest neurite length, and the average number of neurites for each cell. RGCs isolated from embryonic retinas were only stained for βIII-tubulin as RBPMS is not expressed at this developmental time point. For each well, a minimum of 20 RGCs were traced and their data reported.

### RNA extraction, cDNA synthesis, and quantitative reverse transcription-polymerase chain reaction

Total RNA was extracted from RGCs cultured on tissue culture polystyrene plates (TCPS, *n* = 3 each) using TRIzol RNA Isolation Reagent (Life Technologies). Total RNA for each sample (500 ng) was reverse-transcribed into single-stranded cDNA using High-Capacity cDNA Reverse Transcription Kit (Applied Biosystems, Life Technologies), according to the manufacturer’s protocol. PCR reactions were conducted using the prepared cDNA (2 μL) with inventoried TaqMan^®^ Gene Expression Assays (Applied Biosystems, Life Technologies) to detect *GAPDH* (Mm99999915-g1), *Brn3a* (Mm02343791_g1), *Brn3b* (Mm01252278_g1), *Syntaxin1a* (Mm00444008_m1), *PKCalpha* (Mm00440858_m1), *CRX* (Mm00483995_m1), and *Gfap* (Mm01253033_m1). Amplification was performed on a StepOne™ 96-well Real-Time PCR System (Applied Biosystems™). Mixtures were cycled as follows: 50°C for 2 min for one cycle (initial denaturation), one cycle at 95°C for 10 min (denaturing), then 40 cycles at 95°C for 15 s (annealing) and 60°C for 1 min (extension). RT-qPCR reactions were analyzed by calculations of threshold cycle (ΔCT mean) and 2^−ΔCT^ mean. All experimental values were normalized to readings of the endogenous reference gene, glyceraldehyde 3-phosphate dehydrogenase (GAPDH).

### Calcium imaging

Isolated RGCs were seeded onto 35 mm glass-bottom dishes (MatTek Corporation, Ashland, MA, United States) coated with PDL-laminin, using the previously mentioned protocol in this study. 1 × 10^4^ RGCs were seeded per dish and cultured for 7 days in neurobasal-supplemented media before performing calcium imaging experiments. RGCs were washed and stained in extracellular solution (ECS) made in house by supplementing Hyclone Cell Culture-Grade Water (Cytiva, Marlborough, MA, United States) with 137 mM Sodium Chloride, 5 mM Potassium Chloride, 1 mM Sodium Diphosphate, 1 mM Magnesium Sulfate, 10 mM HEPES Buffer, 22 mM D-Glucose and 1.8 mM Calcium Chloride.

Intracellular calcium was stained using FLIPR Calcium 5 Assay Explorer Kit (Molecular Devices, San Jose, CA, United States) reconstituted in ECS as per the manufacturer’s recommended guidelines. Cells were incubated for 15 min in Calcium 5 reagent before washing 1x in ECS. Cells were then imaged at 10x on Leica DMI 6000 fluorescence microscope (Leica, Wetzlar, Germany) using GFP-filter set and analyzed using Molecular Devices Metafluor^®^ Software. Baseline fluorescence was established by recording for 100 s before RGCs were stimulated using a final concentration of either 100 mM potassium chloride or 50 μM glutamate added manually via pipette. Effects of stimulation were recorded for a further 200 s before stopping recording. The analysis was performed by identifying 1 region of interest per cell in each field and background correcting by taking 4 background regions. At least *n* = 10 cells were performed for each condition across at least *n* = 3 experimental dishes. Graphs, traces, and statistical analysis were then produced using GraphPad Prism v9.2 (GraphPad Software, CA, United States) analysis software. Non-calcium supplemented controls were performed using the above method, with the modification of the ECS used not containing 1.8 mM CaCl_2_.

### Statistical analysis

**Figure 1:**
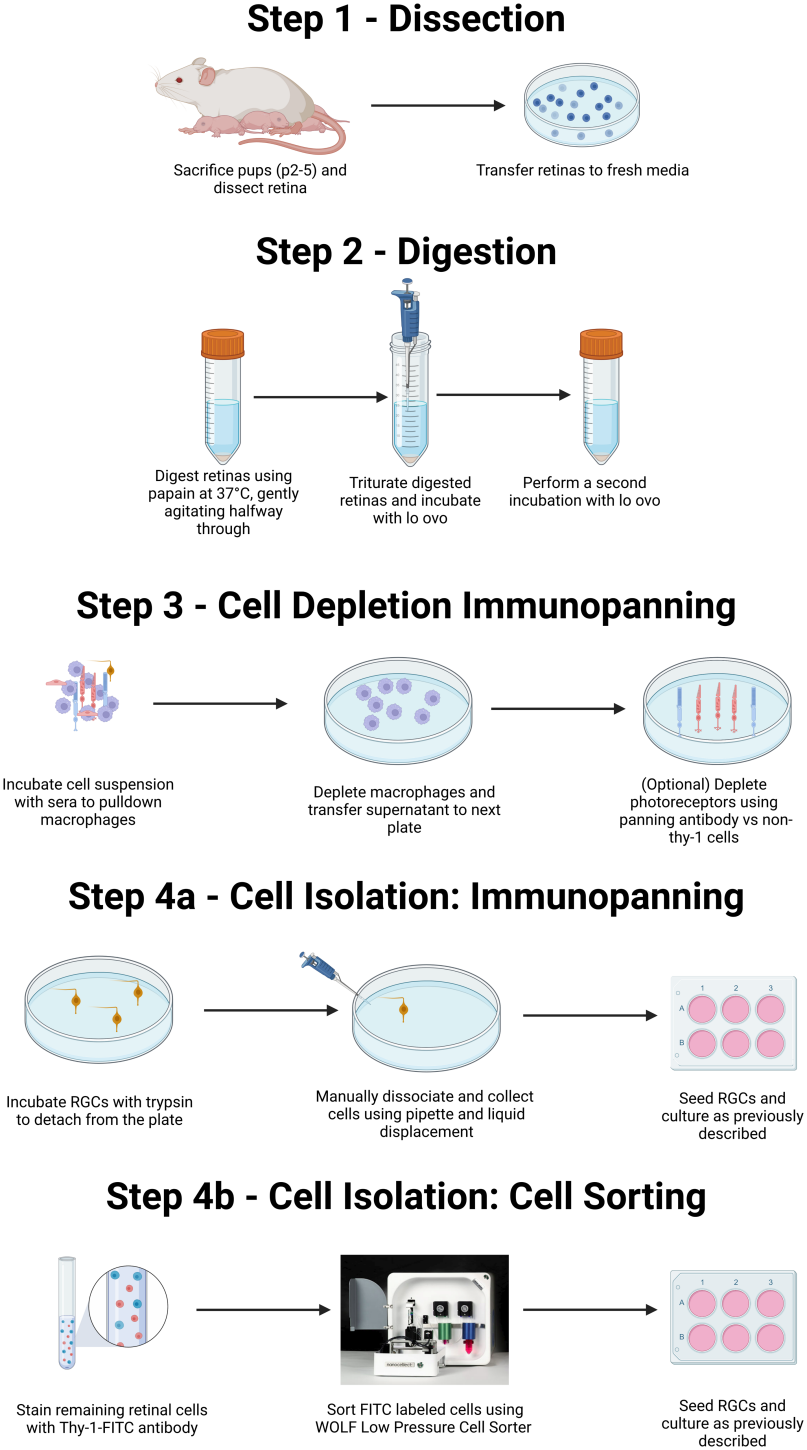
Summary of both immunopanning and low-pressure cell sorting protocols for isolation of RGCs from postnatal rodents. Diagrammatic representation of the isolation of primary RGCs from rodent litters. Both the protocol for immunopanning and protocol for cell sorting will include isolation of retinas from eyes via surgery (Step 1), followed by enzymatic digestion and centrifugation to obtain a single-cell suspension (Step 2). This suspension is then exposed to a series of antibody-coated plates to remove unwanted macrophage, and in some cases photoreceptors (Step 3). The protocol can then proceed either to selection for RGCs, through immunopanning (Step 4A) or via flow-based cell sorting (Step 4B). For purification by immunopanning (Step 4A), cells were immunopanned against Thy-1. Isolated RGCs are then removed via trypsinization in combination with pipetting to facilitate cell release. For RGCs isolated by flowcytometry (Step 4B), the retinal suspension can be incubated with a fluorescently labeled Thy-1 antibody before being sorted using a low-pressure cell sorter. Created with BioRender.com.

All experiments were performed three times unless otherwise stated. Statistical analysis was performed using GraphPad Prism Software 9.2 following confirmation of normal data distribution. All data were presented as the mean ± standard deviation. Two groups were compared using unpaired Student’s *t*-test, and multiple groups were compared using one- or two-way ANOVA with *post-hoc* Dunnett’s test. A value of *p* ≤ 0.05 was considered significant (Figure 1).

## Results

### Cell survival, morphology, and outgrowth

**Figure 2:**
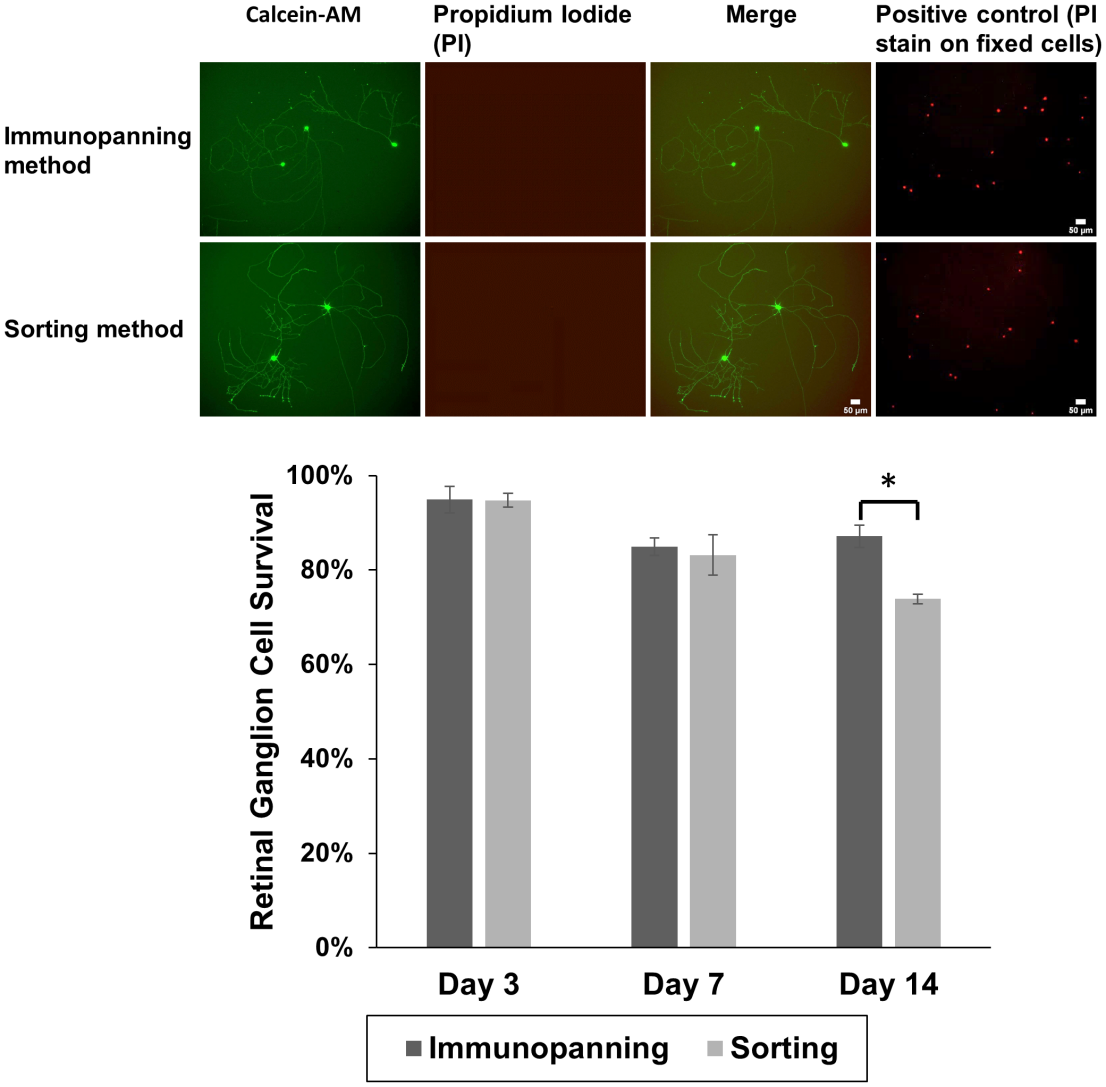
Representative fluorescence microscopy images showing attachment and neurite extension of RGCs purified by immunopanning method and sorting method using Cellstain™ staining to show green fluorescent viable cells (calcein AM-stained) and red fluorescent dead cells (propidium iodide, PI), over 3 days at 37°C. By day 3, cells had adopted a more spread RGCs morphology. Positive control images (PI stain on fixed cells from a separate sample cultured from the same experiment) are shown. RGC viability was calculated for both isolation methods after 3, 7, and 14 days. The data is reported as the mean ± SD, significance was determined by Student’s *t*-test *p* < 0.05. (Scale bar = 50 μm).

Following 3 days in culture, the viability of RGCs purified from each method was evaluated. RGCs were stained with the Cellstain™ double-staining kit using Calcein-AM to label live cells fluorescently in green and dead cells with propidium iodide in red (Figure 2). Notably, RGCs purified by the sorting method remained viable with no cytotoxicity evident and similarly extended their neurites to those purified by the immunopanning method after 3 days in culture. There was also little evidence of dead cells on cultured wells with viability of approximately 95% from both methods following 3 days in culture, and the axon/dendritic morphology of RGCs was maintained. Some cell death was observed in both isolation methods at later time points, as well as significant levels of neurite degeneration. A significant difference was observed in the survival after 14 days in culture, however even at this time 73.9% of attached cells from the sorting method remaining viable.

**Figure 3:**
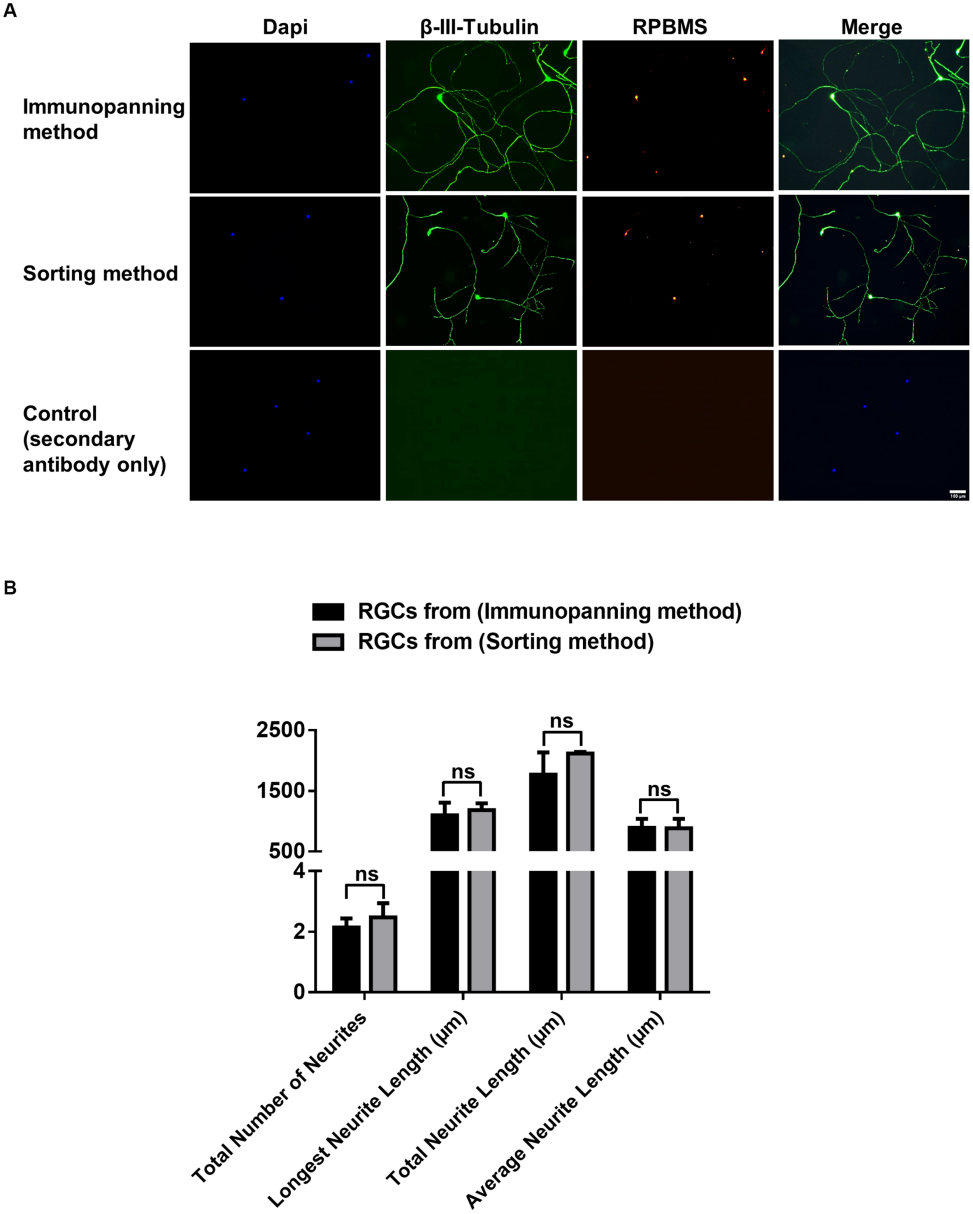
**(A)** Representative fluorescence microscopy images showing effects of immunopanning method and sorting method as RGCs purification techniques on neuronal and RGCs markers. Immunocytochemical staining was performed for βIII-tubulin (green), RPBMS (red), with DAPI nuclear counterstain (Blue), after 3 days of culture. Negative control images are shown (scale bar = 100 μm). **(B)** Graphs showing tracing data for RGCs obtained from the immunopanning method and sorting method with a minimum of 20 cells traced per well. The total number of neurites/cell, longest neurite length/cell, total neurite length/cell, and average neurite length/cell were calculated. The values were reported as the mean ± SD of three independent experiments: *n* = 3 in triplicates. Statistical significance was determined by Student’s *t*-test: (ns) *p* > 0.05.

Immunocytochemistry was conducted for cells cultured on 24-glass-bottom well plates purified by immunopanning and sorting methods to identify the expression of RGC associated markers RNA-Binding Protein with Multiple Splicing (RPBMS) and βIII-tubulin (Figure 3A). βIII-tubulin and RPBMS-positive staining were observed in all cultured cells, indicating that both methods maintained the RGCs phenotype. Furthermore, neurite outgrowth and extension were evaluated by measuring the total number of neurites, longest neurite length, total neurite length, and the average neurite length for RGCs purified from both methods (Figure 3B). Immunopanning and sorting methods resulted in a total number of neurites of 2.14 ± 0.3 and 2.47 ± 0.5, longest neurite length of 1098.14 ± 205.5 μm and 1184.15 ± 111.3 μm, total neurite length of 1765.5 ± 367.7 μm and 2117.6 ± 23.3 μm and the average neurite length of 890.9 ± 148.9 μm and 882.2 ± 157.1 μm, respectively. These results showed no significant difference (*p* > 0.05) between RGCs obtained by either method. This finding indicates that the sorting method has no adverse effect on RGCs neurite outgrowth and extension compared to the immunopanning method.

**Figure 4:**
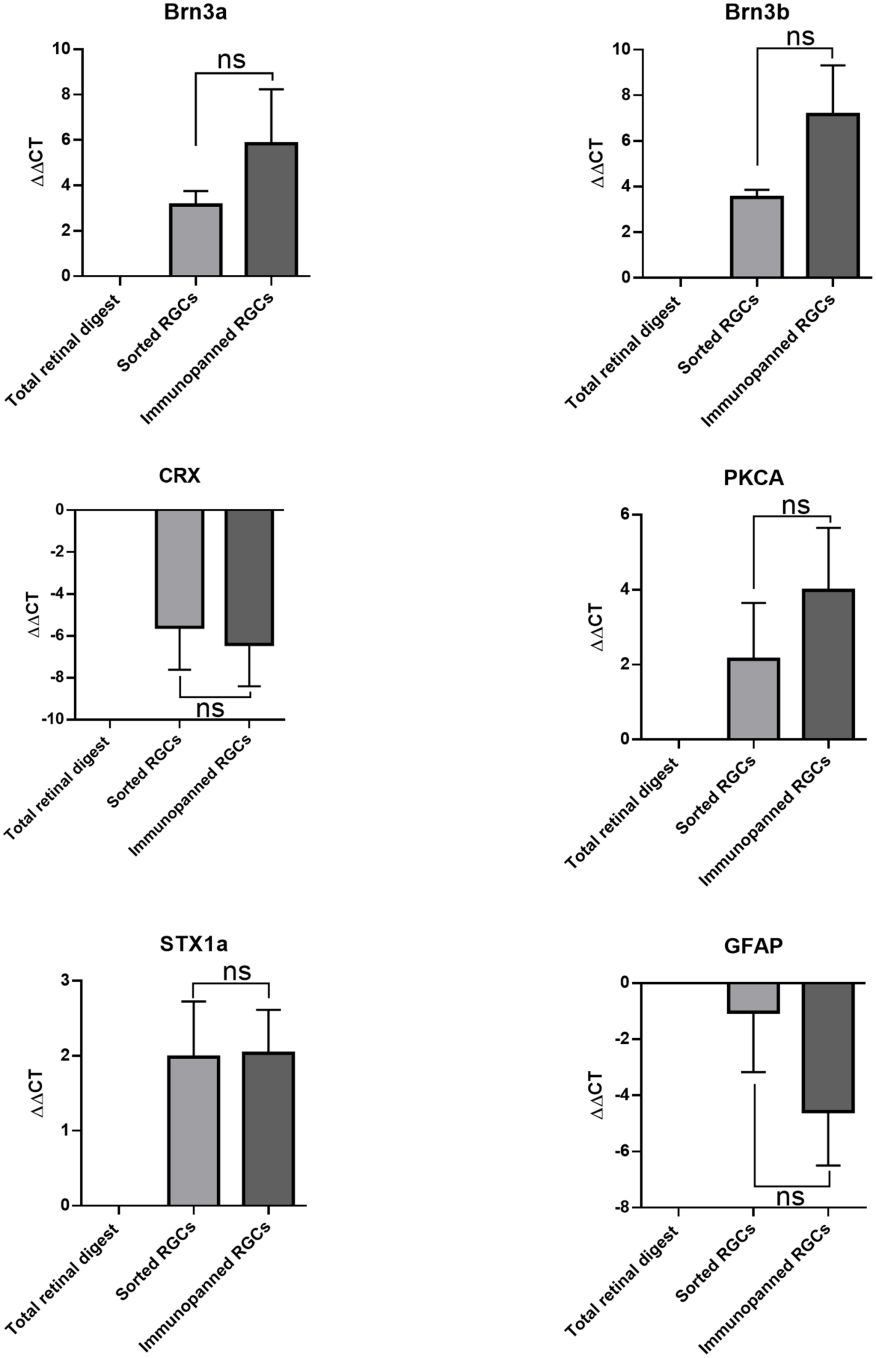
Comparative effects of purification methods on gene expression of RGCs. ∆∆CT was determined by RT-qPCR for *Brn3a*, *Brn3b*, *CRX*, *PKCA*, *STX1a*, and *GFAP*. Expression is normalized to *GAPDH* and represented relative to total retinal digest (mean ± SEM of three independent experiments: *n* = 3 in duplicates). Statistical significance versus total retinal digest (one-way ANOVA): (ns) *p* > 0.05.

qRT-PCR was carried out to identify the retinal cell types collected in each purification method. Isolated mRNA was tested for *Brn3a and Brn3b* (RGCs markers) (Badea et al., 2009), *Syntaxin1a* (amacrine cell marker) (Jin et al., 2015), *CRX* (photoreceptor marker) (Hennig et al., 2008), *Gfap* (glia marker) (Sarthy et al., 1991), *and PKCalpha* (bipolar cell marker) (Haverkamp et al., 2003; Figure 4). There were no significant differences in expression levels between cells isolated via immunopanning when compared to low-pressure FACS sorting. The data showed that both isolation methods promoted the isolation of RGCs, with increased expression of *Brn3a* and *Brn3b* relative to the total retinal digest. In contrast, gene levels associated with photoreceptor and glia cells *CRX and Gfap* were decreased relative to total retinal digest in both methods. *Syntaxin1a* and *PKCalpha* expression level both increased relative to total retinal digest, however this increase could be due to a small number of contaminating cells being present but in a much smaller total number of cells, as RGCs make up 2.5% of the total cells in the retina (Young, 1985).

**Figure 5:**
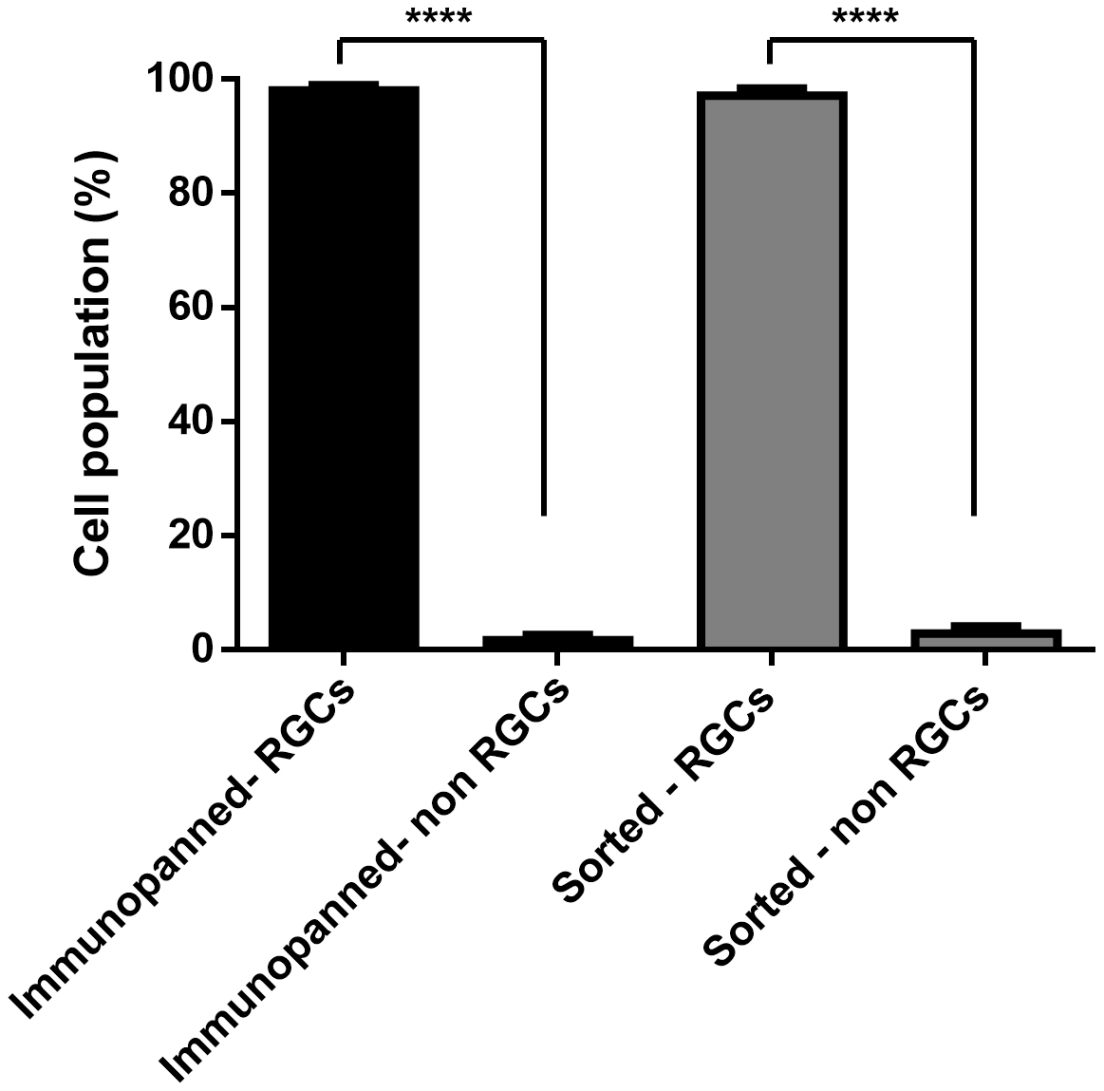
Percentage of living cells staining positive or negative for the RGC marker RBPMS for both immunopanning and IP-FACS sorted cells. The values were reported as the mean ± SD of three independent experiments: *n* = 3 in triplicates. Statistical significance RGCs versus non-RGCs (Student’s *t*-test): (****) *p* < 0.0001.

To further confirm the purity of both methods and determine the level of contamination present of other cell types, immunostaining was conducted. DAPI signals associated with RGCs stained with RPBMS and DAPI signals not associated with RGCs were counted. Cell counts, shown in Figure 5, indicate that both methods were not significantly different and resulted in 98.08% ± 2.5% immunopanned RGCs and 97.13% ± 3.7% sorted RGCs. Furthermore, both methods resulted in a significant difference between RGCs and non-RGCs (*p* < 0.0001).

### Calcium imaging results

**Figure 6:**
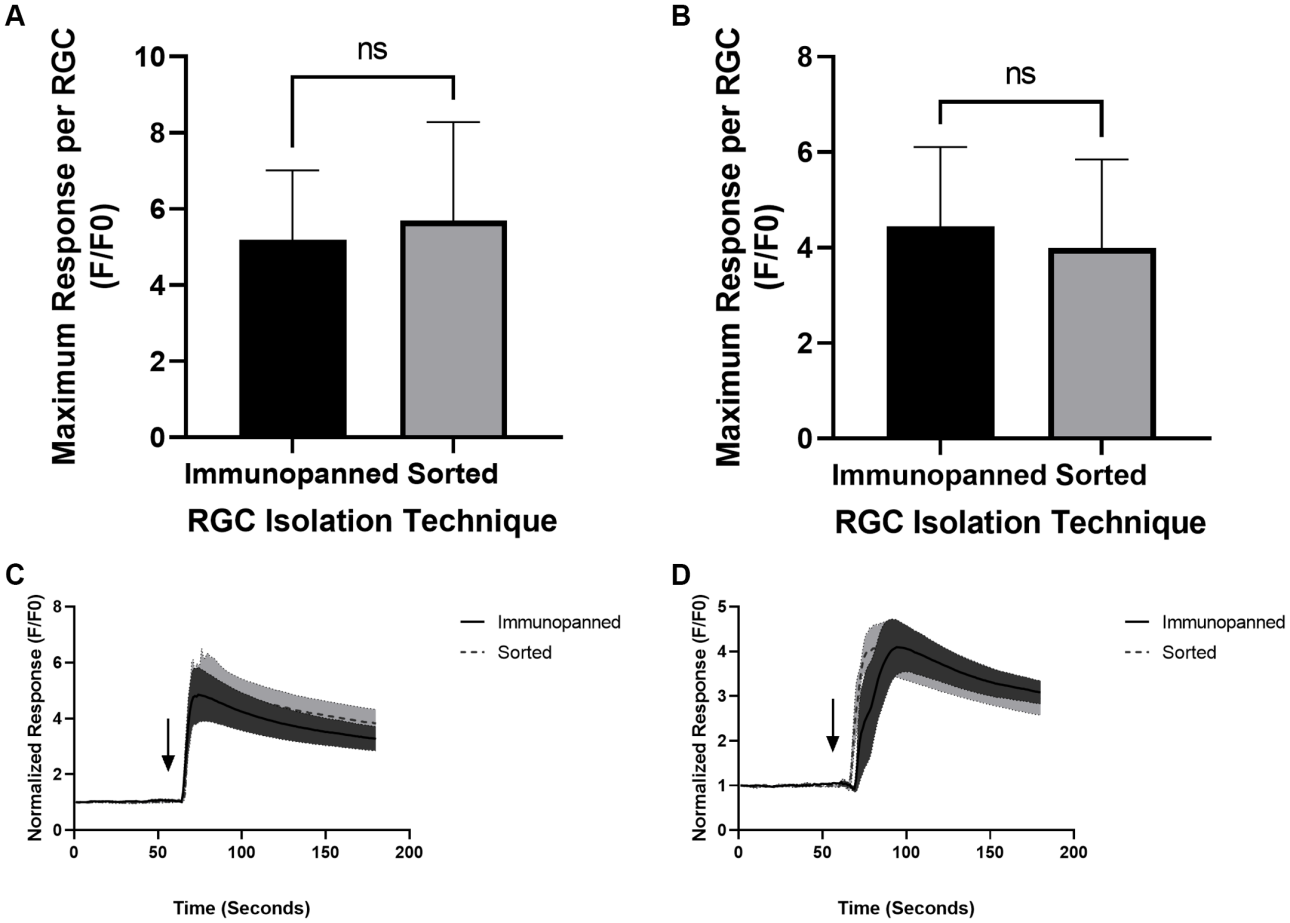
RGCs respond to calcium-associated stimuli regardless of isolation technique. **(A,C)** RGCs depolarize in response to 100 mM KCl stimuli and **(B,D)** 50 μM glutamate stimuli. Baseline activity was established over 60 s before the stimulus was applied to cells via a manual pipette (addition of stimulus indicated on traces via an arrow). The maximum response between conditions was non-significant. Original videos are included in Supplementary material.

*In vivo*, RGCs respond to light by opening plasma membrane voltage-gated Ca^2+^ channels with a corresponding increase of intracellular calcium, generating an action potential that is transmitted to the brain (Gartland and Detwiler, 2011); while *in vitro*, RGCs can be directly simulated by small molecules. Here, we use two different molecules: potassium chloride, which in high concentrations is known to cause an influx of Ca^2+^ into neurons, and glutamate, an excitatory neurotransmitter secreted by bipolar cells *in vivo* which increases cytosolic calcium by activating NMDA and AMPA plasma membrane receptors of the RGCs (Yang, 2004; Hartwick et al., 2008). RGCs which were both immunopanned and sorted showed no significant difference in their response to both potassium chloride (Figures 6A,C) or glutamate (Figures 6B,D). RGCs were also unresponsive to either stimulus in calcium-free controls (data not shown), indicating that extracellular calcium must be present for stimulation to occur. Videos of pre- and post-stimulated RGCs are available in ">Supplementary Videos S1–S4.

### Isolation of embryonic retinal ganglion cells

**Figure 7:**
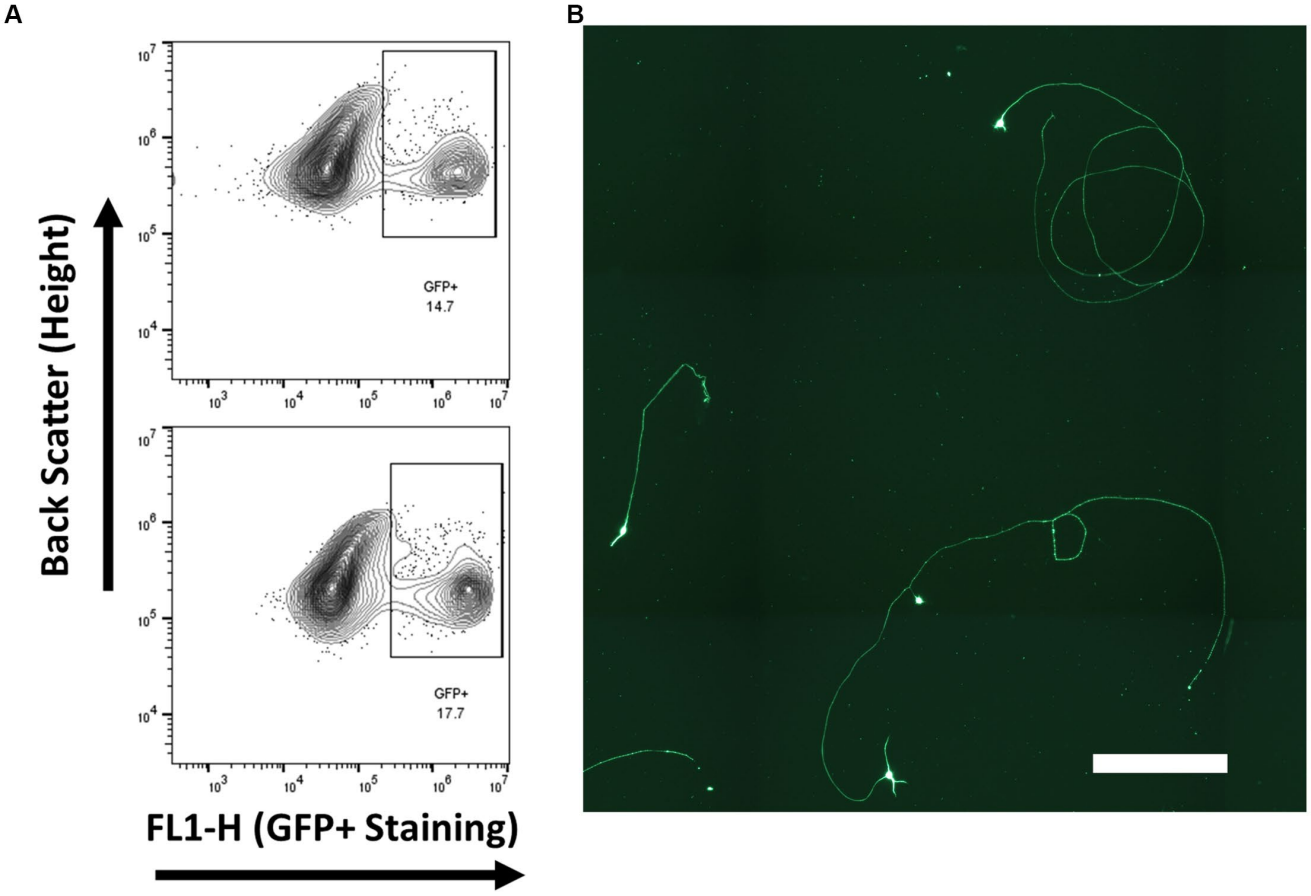
RGCs were isolated from Brn3b-P2A-eGFP at embryonic day 14–15 and purified via low-pressure flowcytometry. **(A)** Gating conditions for the purification of RGCs. **(B)** Staining of isolated cells with βIII-tubulin following growth for 2 days *in vitro.* (Scale bar 250 μm).

Retinal ganglion cells from embryonic day 14–15 via endogenous Brn3b promoted eGFP expression. Gated GFP cells composed approximately 15% of the total cell population (Figure 7) and were cultured on tissue culture plates coated with PDL and laminin. RGC cell identity was confirmed by βIII-tubulin expression rather than with RBPMS or Thy-1, as both of these markers are not expressed at this developmental age point. All living cells, cells without a contracted DAPI signal, were observed to be βIII-tubulin positive.

## Discussion

Purified cultures of retinal ganglion cells have been a vital tool in the understanding of retinal cell biology, axon growth, the identification of neurite guidance factors and the testing of neuroprotective agents for the treatment of glaucoma and other neurodegenerative diseases. The isolation of RGCs at different developmental time points has been used in the identification and screening of different intrinsic factors and microRNAs that help to govern axon regeneration and which may play an important role in future treatments of diseases of the retina and for spinal cord injury (Wang et al., 2007; Mead et al., 2020). More recently, with the success demonstrated in photoreceptor transplantation, RGCs purified using the immunopanning technique have been used for rodent *in vivo* transplantations which have shown dendritic integration and electrophysiological responses to light stimulation (Venugopalan et al., 2016). Still, in photoreceptor transplantation it was shown that the age and maturity of the photoreceptors had an effect on the ability of the transplanted cells to integrate into the retina (Gonzalez-Cordero et al., 2013; Tezel and Ruff, 2021). While embryonic RGCs have shown an increased potential for neurite outgrowth (Steketee et al., 2014), these cells cannot be isolated using the currently developed immunopanning or magnetic separation methods as these methods rely on the expression of the surface antigen Thy-1 which is not expressed during the initial stages of RGC development. The development of the CRISPR-Cas gene editing mechanism has led to the development of new optogenetic tools, including mouse and stem cell models in which RGCs and their subtypes are labeled from their initial development but which require the use of FACS sorting methods, which have been shown to be toxic to sorted RGCs (Miltner et al., 2019).

Using the immunopanning method for comparison, we tested the use of low-pressure cell sorting, a system designed for use with pressure sensitive cells, on the isolation of RGCs by isolating cells from the same animals by using a fluorescently labeled antibodies against Thy-1. In this way, we were able to expose the cells to exactly the conditions during dissection and during the mechanical disassociation of the retinal cells and ensure the same population of cells would be isolated in both techniques. Using the low-pressure sorting system, we found that both the purity as determined through immunostaining for the RGC marker RBPMS and the 3 and 7 days *in vitro* survival of RGCs was not significantly different when compared to the RGCs from the immunopanning method, though differences were observed in the expression levels of RGC specific genes when evaluated by qPCR. We sought to further evaluate the health of the isolated RGCs and their potential for being used in transplantation experiments by examining their outgrowth and their function using calcium imaging. In outgrowth assays we saw no difference in either the average and longest neurite length of the RGCs or in the total number of neurites greater than two cell bodies in length. During calcium imaging experiments, RGCs from both groups were observed to depolarize both in response to potassium chloride and glutamate, demonstrating that the RGCs are capable of not just producing an action potential but also depolarizing in response to the correct physiological stimuli.

Low-pressure cell sorting does have drawbacks, the largest being the time necessary for isolation. Because low = pressure cell sorters operate at a much slower rate of approximately 300 cells per second in comparison to approximately 10,000 cells per second for traditional high pressure cell sorting, purifying cells, especially cells in a low concentration such as the RGCs, can take several hours in order to obtain a largest enough number to conduct experiments. While we did verify that cells purified up to 6 h later were still viable, we did not investigate whether RGCs purified later during sorts expressed different gene profiles than those cells purified earlier. In order to increase the efficiency of the sort, we combined the sorting method with an immunopanning step to remove CD73+ cells, a population which includes immature photoreceptors, to increase the isolated RGC population from 1.6 to 2.6% of the total population. The identification of surface markers for other retinal cell types may provide a way to further enrich the RGCs within the cell population to be sorted, ultimately producing a faster overall purification time (Supplementary Figure S1). Additionally, this combining of immunopanning and low-pressure cell sorting could prove a useful method for the studying of individual RGC cell subtypes by first isolating all RGCs using the established immunopanning method, followed by purification of the individual subtypes via already established optogenetic labels.

This drawback was not as relevant for early embryonic RGC purifications, as the RGCs make up a substantially higher percentage of the cells in the developing retina, with approximately 15% of the sorted cells expressing the Brn3b promoted GFP marker. It would be expected that this ratio of RGCs would be conserved should the cells be isolated from a retinal organoid, the cell source currently used for photoreceptor transplantations, which mimics the developmental timeline of the native retina.

It should be noted that most assays in this study demonstrate the isolation of RGCs from rats, following the initial immunopanning methods established by Barres et al., while the sorting of the embryonic RGCs were isolated from transgenic Brn3b-P2A-eGFP mice, which are not available in a rat model. It is not expected that this difference in species will have an effect of the viability or health of RGCs isolated using this method, as immunopanning has been demonstrated for the purification of RGCs from rats, mice, chicks and humans, though some properties may vary based upon the subset of RGCs which react to the antibody used (Barres et al., 1988; Butowt et al., 2000; Zhang et al., 2010; Winzeler and Wang, 2013). Overall RGCs from both rats and mice demonstrate similar reactivity to established RGC markers (Nadal-Nicolás et al., 2023).

In conclusion, low-pressure FACS can be used to isolate RGCs from early postnatal rodents with comparable phenotype and function to the standard of immunopanned RGCs. We have further demonstrated that this method can be used for the isolation of embryonic RGC populations which cannot currently be purified by combining FACS isolation with fluorescently labeled transgenic animals. This technique provides a new important tool for the study of early born RGCs, the studying of RGC subtypes and for creating cell populations for cell transplantation.

## Data availability statement

The original contributions presented in the study are included in the article/Supplementary material, further inquiries can be directed to the corresponding author.

## Ethics statement

The animal study was reviewed and approved by the Institutional Animal Care and Use Committee of the University of Missouri-Kansas City.

## Author contributions

KM and AA contributed to the experiment design, data collection, data analysis, writing, and figure preparation. AP contributed to the experiment design, data collection, and analysis for Figure 6. AB contributed to the data collection and analysis of Figures 2, 5. SN-G contributed to the data collection and analysis of Figures 3, 7. KK contributed to the experimental design, oversaw experimentation, and contributed to figure preparation and writing. All authors contributed to the article and approved the submitted version.

## Funding

This work was supported by the National Eye Institute (R01EY028946) and from the School of Medicine of the University of Missouri-Kansas City. Research reported in this publication was supported in part by a grant from the National Center for Research Resources and National Institute of General Medical Sciences (RR027093) of the National Institutes of Health. Additional support by the Felix and Carmen Sabates Missouri Endowed Chair in Vision Research was gratefully acknowledged.

## Conflict of interest

The authors declare that the research was conducted in the absence of any commercial or financial relationships that could be construed as a potential conflict of interest.

## Publisher’s note

All claims expressed in this article are solely those of the authors and do not necessarily represent those of their affiliated organizations, or those of the publisher, the editors and the reviewers. Any product that may be evaluated in this article, or claim that may be made by its manufacturer, is not guaranteed or endorsed by the publisher.

## Supplementary material

Supplementary Figure 1 Effect of photoreceptor depletion on RGC isolation. **(A)** Unstained control of retinal cell population was used to set baseline gates for identifying Thy-1 positive RGCs. **(B)** Representative image of retinal cell suspension before immunopanning step. Cells in this group did not undergo photoreceptor depletion. **(C)** Representative image of retinal cell suspension before immunopanning, but after photoreceptor depletion panning step. This resulted in a modest increase in the RGC % of the retinal cell population.

Supplementary Videos Sample recordings of calcium response experiments whose results are reported in Figure 6. Recordings for RGCs isolated using the immunopanning method and the cell sorting method show calcium response to the injection of either 50 μM glutamate or 100 mM KCl stimuli following 60 seconds of baseline recording. Samples were conducted as batch experiments and calcium response was measured using the FLIPR Calcium 5 fluorescent dye.
