# Supplementary figures and images for: Purification of retinal ganglion cells using low-pressure flow cytometry
Source: Front Mol Neurosci. 2023 Jul 20;16:1149024. doi: 10.3389/fnmol.2023.1149024 (PMC10400357; doi:10.3389/fnmol.2023.1149024)

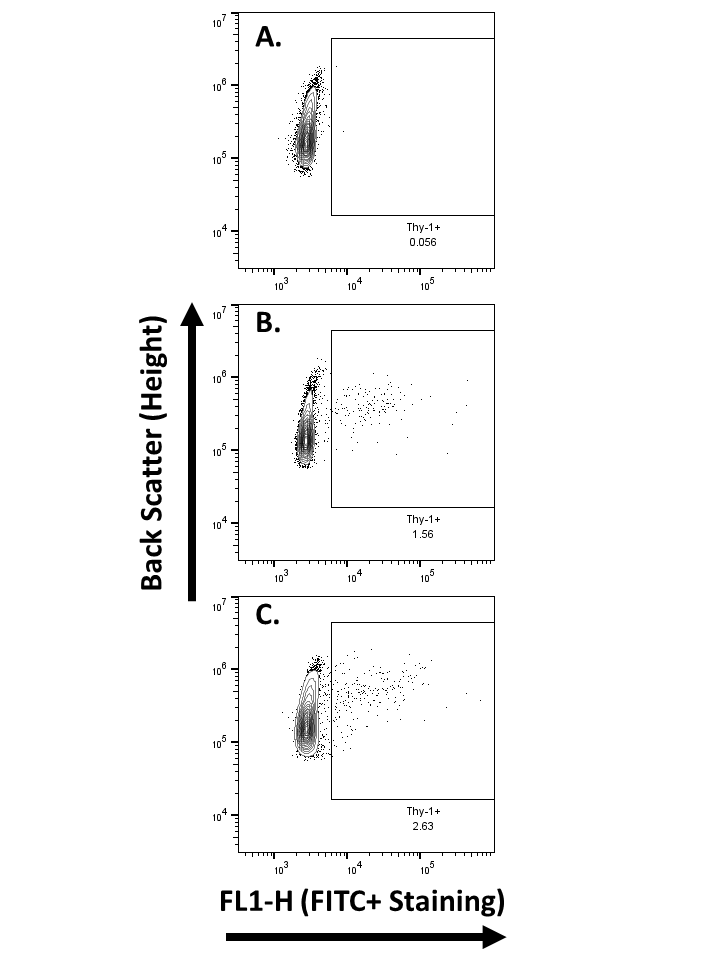

Supplement: Supplementary Figure 1 — Effect of photoreceptor depletion on RGC isolation. (A) Unstained control of retinal cell population was used to set baseline gates for identifying Thy-1 positive RGCs. (B) Representative image of retinal cell suspension before immunopanning step. Cells in this group did not undergo photoreceptor depletion. (C) Representative image of retinal cell suspension before immunopanning, but after photoreceptor depletion panning step. This resulted in a modest increase in the RGC % of the retinal cell population. [file Image_1.TIF]
